# G protein-coupled estrogen receptor (GPER) modulates epithelial-mesenchymal transition-like phenotype in human glioblastoma cells

**DOI:** 10.1080/19336918.2026.2720325

**Published:** 2026-08-21

**Authors:** Karla Mariana Peña-Gutiérrez, Karina Hernández-Ortega, Beatriz Arantxa Castro-Reyes, Andrea Rosas-Benitez, Edgar Ricardo Vázquez-Martínez, Rocío García-Becerra, Ignacio Camacho-Arroyo

**Affiliations:** a Unidad de Investigación en Reproducción Humana, Instituto Nacional de Perinatología-Facultad de Química, Universidad Nacional Autónoma de México, Mexico City, México; b Facultad de Química, Departamento de Biología, Universidad Nacional Autónoma de México, Mexico City, Mexico; c Departamento de Biología Molecular y Biotecnología-Programa de Investigación de Cáncer de Mama, Instituto de Investigaciones Biomédicas, Universidad Nacional Autónoma de México, Mexico City, Mexico

**Keywords:** 17β-estradiol (E2), brain cancer, epithelial-mesenchymal transition-like, G-1, G-15, glioblastoma, GPER, invasion, migration

## Abstract

Glioblastomas (GBs) are the most frequent and aggressive central nervous system tumors, whose progression is influenced by sex hormones. 17β-estradiol (E2), promotes an epithelial-to-mesenchymal transition (EMT)-like process and enhances proliferative, migratory, and invasive capacities in GB through intracellular estrogen receptors. However, the role of G protein–coupled estrogen receptor (GPER) in GB remains poorly understood. We evaluated the effects of E2, the GPER-agonist G-1, and the GPER-antagonist G-15 on EMT-related processes, migration, and invasion in U251 and U87 GB-derived cells. GPER activation by E2 and G-1 increased migration and invasion, whereas G-15, alone or combined, reduced these effects. GPER activation also induced a mesenchymal-like phenotype. These findings suggest that GPER-associated signaling contributes to EMT-like phenotypic changes, migration, and invasion in GB.

## Introduction

Gliomas are the most common primary malignant tumors of glial origin within the central nervous system (CNS) [1]. They are classified into four grades according to their degree of malignancy, with grade IV tumors being the most aggressive [2,3]. Glioblastoma (GB), a grade IV glioma, is the most common and aggressive CNS tumor, accounting for approximately 60% of cases and characterized by high proliferative and invasive capacities, as well as frequent recurrence [1,4]. GB has been classified into three molecular subtypes: a) Proneural (PN), associated with better survival [5,6]; b) Classic (CL), which exhibits astrocyte characteristics [6,7]; and c) Mesenchymal (ME) subtype, linked to a poorer prognosis for survival [7,8].

However, the cellular and molecular processes involved in GB progression have not been fully described, including the mechanisms that generate distinct cellular phenotypes. Among these, an epithelial-mesenchymal transition-like (EMT)-like phenotype has been proposed to play a crucial role in the invasive capacity of GB [9–11]. EMT is a cellular reprogramming process in which epithelial cells lose intercellular junctions and acquire a mesenchymal phenotype with enhanced migratory and invasive capabilities [12].

EMT is a reversible process in which mesenchymal cells can reacquire the epithelial characteristics through mesenchymal-to-epithelial transition (MET) [13]. Although the CNS lacks epithelial tissue, several invasive mechanisms overlap between CNS neoplasms and other cancers, since factors that induce EMT in epithelial tumors can also promote mesenchymal features in GB cells [14].

Estrogens, especially 17β-estradiol (E2), have been reported to regulate GB growth through binding to intracellular estrogen receptors (ERα or ERβ), thereby inducing the expression of genes involved in cell malignancy. E2 has also been observed to induce an EMT-like phenotype by altering cell morphology and cytoskeletal organization, consequently enhancing the migratory and invasive capacities of GB cells. Therefore, E2 has been proposed as a contributor to GB progression [15–17].

In several neoplasms, including breast, ovarian, endometrial, and prostate cancers, the G-protein-coupled estrogen receptor (GPER) has been shown to bind E2 and rapidly and transiently activate numerous intracellular signaling pathways, thereby contributing to cancer cell proliferation, migration, and invasion [18–20].

In the context of GB, GPER protein expression has been reported in the human GB-derived cell lines U251 and LN229 [21]. In a previous study from our group, GPER expression at both the mRNA and protein levels was determined in U251, U87, LN229, and T98G human GB-derived cell lines. Moreover, GPER was localized at the cell membrane and in the cytoplasm, and the U87 cell line exhibited the highest GPER expression among the analyzed cell lines. Additionally, E2-mediated autoregulation of GPER expression was observed [22]. However, the role of this receptor in GB progression is unknown. Therefore, in the present study, we investigated the contribution of GPER to EMT-like phenotype, migration, and invasion in human GB-derived cell lines.

## Materials and methods

### Bioinformatic analysis

To determine the expression profile of GPER in GB, an initial *in silico* analysis was performed using The Cancer Genome Atlas (TCGA) datasets for low-grade gliomas (LGG, *n* = 534) and GB (GBM, *n* = 391). These data were compared with normal (non-tumor) brain tissue samples (*n* = 2931) obtained from the Genotype-Tissue Expression (GTEx) portal (https://gtexportal.org/home/). Subsequently, the expression profiles of GPER, epithelial markers (SOX2 [SRY-box transcription factor 2] and TJP1 [ZO-1]), and mesenchymal markers (VIM [vimentin] and CDH2 [N-cadherin]) were evaluated across the different molecular subtypes of GB using TCGA-GBM data. All analyses were performed in RStudio using GDCQuery for RNA-Seq gene expression data. The following R packages (4.4.3) were used to obtain transcriptomic profile data from gene expression quantification [23]: BiocManager, TCGAbiolinks, GEOquery, SummarizedExperiment, DESeq2, biomaRt, ggplot2, ggsignif, and multcomp. Graphs and statistical analyses were generated using ggplot2 and ggsignif, while multcomp was used for post hoc comparisons. A statistical difference was established for values of *p < .05, **p < .01, ***p < .001, ****p < .0001, with a log2 fold change >1.

### Cell culture

Cell lines U251-MG from a biopsy of a 61-year-old Caucasian man and U87-MG (HTB-14, glioblastoma of unknown origin) were purchased from ATCC. The U251 and U87 cells were previously authenticated by the authors using STR profiling with the AmFISTR® IdentifilerTM kit (Cat 4,322,288) and the Genetic Analyzer 3130 × 1 (Applied Biosystems; Thermo Fisher Scientific, Inc). Cells were cultivated to 25–30 passages, assessed for the preservation of morphological characteristics, and routinely monitored for mycoplasma contamination by microscopy (data not shown). Cell lines were cultured in DMEM supplemented with phenol red (Biowest, S.A., USA), 10% fetal bovine serum (FBS, Biowest), 1 mM sodium pyruvate (Biowest, S.A., USA), 0.1 mM non-essential amino acids (Invitrogen, USA), and 1 mM antibiotic-antimycotic solution (streptomycin 10 g/L, penicillin G 6.028 g/L, and amphotericin B 0.025 g/L, L0010). Cells were incubated under standardized conditions of 37°C, 95% air, 5% CO2, and 95% humidity.

### Treatments

When cells reached 70–80% confluence, the culture medium was changed to DMEM without phenol red (Invitrogen, USA) supplemented with 10% charcoal/dextran treated FBS (C-FBS, Biowest), 0.1 mM non-essential amino acids (Invitrogen, USA), and antibiotic-antimycotic solution (streptomycin 10 g/L; penicillin G 6.028 g/L; and amphotericin B 0.025 g/L, L0010) and incubated for 24 h. *Standardization*: Cells were treated with 17β-estradiol (E2, Cat. E4389, Millipore Sigma) 10 and 100 nM; E2 vehicle (CDX = cyclodextrin 0.01%, Cat. C-0926, Millipore Sigma); G-1 (GPER-specific agonist, 1-(4-(6-Bromobenzo1,3dioxol-5-yl)-3α,4,5,9β-tetrahydro-3 H-cyclopenta[c]quinolin-8-yl)-ethanone, Cat. 3577, Tocris) 10 and 100 nM; G1 vehicle (DMSO 0.01%, Cat. D2650, Sigma-Aldrich); G-15 (GPER-specific antagonist, (3αS,4 R,9βR)-4-(6-bromo-1,3-benzodioxol-5-yl)-3α,4,5,9β-tetrahydro-3 H-cyclopenta[c]quinoline, Cat. 3678, Tocris) 1, 5, and 10 µM; and its vehicle (DMSO 0.01%). After the initial experiments, we used E2 [10 nM], G-1 [10 nM], and G-15 [5 and 10 µM]. For combined treatments, cells were pretreated with G-15 for 30 min before the addition of E2 or G-1; the treatments were renewed every 24 h.

The initial concentration ranges were selected based on previously published studies and the reported binding affinities of E2, G-1, and G-15 for GPER [24–26]. Preliminary dose-response experiments (Supplementary Figures S1–5) were performed to determine the concentrations that produced reproducible biological effects. Based on these results, E2 (10 nM), G-1 (10 nM), and G-15 (10 µM for U251 cells and 5 µM for U87 cells) were selected for the subsequent functional assays.

### Cell viability determination by trypan blue exclusion

A total of 1 × 10^4^ cells were seeded in 12-well plates. After treatment, cells were washed twice with 1X PBS (NaCl 137 mM, KCl 2.7 mM, KH_2_PO_4_ 1.8 mM, Na_2_HPO_4_ 10 mM, pH 7.4) and detached with PBS-EDTA (1.2 mM). Cells were collected into 1.5 mL microcentrifuge tubes and centrifuged at 2,500 rpm for 1 min. The supernatant was discarded, and the cell pellet was resuspended in 1 mL of DMEM with phenol red. After this, 10 µL of 0.2% trypan blue solution was added to the cell suspension at a 1:1 ratio, and 10 µL of the mixture was added to each well of the Neubauer chamber [27]. Viable, non-viable, and total cells were counted at 24–96 h using a Countess™ II automated cell counter (Invitrogen, Thermo Fisher Scientific; AMQAX1000). Cell viability was calculated using the following formula:

$$\mathrm{Cell}\;\mathrm{viability}\;\left(\%\right)=\frac{\mathrm{Viable}\;\mathrm{cells}}{\mathrm{Total}\;\mathrm{cell}\left(\mathrm{Viable}+\mathrm{non}-\mathrm{viable}\;\mathrm{cells}\right)}\times 100$$

Cell viability was expressed as the percentage of viable cells.

### Cell migration assay

Wound-healing assays were used to determine the migratory capacity of U251 and U87 cells, seeded at 2 × 10^5^ cells per well in 6-well plates. Before treatment, a wound was made in the cell monolayer using a 200 µl pipette tip. Cells were then washed with 1X PBS, and DMEM without phenol red was added in conjunction with a 1 h pretreatment with the selective DNA synthesis inhibitor cytosine β-D-arabinofuranoside hydrochloride (Ara-C; 10 µM; Cat. No. C1768, Sigma-Aldrich) [28,29] to minimize the contribution of cell proliferation to wound closure and ensure that the observed effects primarily reflect changes in cell migration. Images of the wound area were captured at 100x total magnification using an inverted microscope (CKX41 Olympus) coupled to an Infinity 1–2C camera (Lumenera, Ottawa, Ontario, Canada). Four random fields were captured for each experimental condition at 0, 12, 24, and 48 h after treatment. Wound closure was analyzed using the MRI Wound Healing Tool plugin for ImageJ [30].

### Invasion assay

For the U251 and U87 cell invasion assays, a modified Boyden chamber was used. A Transwell insert containing a 10 µm-thick, 8.0 µm pore-sized porous polycarbonate membrane (Cat. No. 3422, Corning, New York, USA) was installed. On this membrane, a 2 mg/mL gel of extracellular matrix proteins derived from Engelbreth-Holm-Swarm murine sarcoma cells (Cat. No. E1270, Matrigel, Sigma-Aldrich) was prepared in DMEM medium without phenol red or FBS. This gel was incubated at 37°C for two h. Once the 50 µL of matrigel was formed, 2 × 10^4^ cells were seeded in DMEM medium without FBS, but with Ara-C (10 μM), and the treatments of interest were placed in the upper chamber. DMEM medium containing FBS and a chemoattractant was placed in the lower compartment. The cells were then incubated at 37°C with 5% CO_2_ for 24 h. Then, the culture medium and Matrigel were removed from the inserts. Two washes with 1X PBS were performed. The cells that had invaded the polycarbonate membrane were fixed with 2% paraformaldehyde (PFA) for 20 min at room temperature, followed by three 5 min washes with PBS under moderate agitation. Cells were stained with 1% crystal violet for 20 minutes, washed five more times with PBS, and left to dry for 24 h. The polycarbonate membranes were cut from the inserts, mounted on coverslips with histological mounting medium (DPX Mountant for Histology, Cat. No. 06522, Sigma-Aldrich) and left to dry for 24 h. Finally, the invasive cells were observed under a microscope (Olympus CKX41) at 100X total magnification, and images were obtained with a Lumenera Infinity 1–2C camera. Six random fields were captured, and the images were analyzed using the Cell Counter plugin in ImageJ software.

### Morphology analysis test (fractal analysis)

The geometric characteristics of the treated cells were observed using phase contrast microscopy at 400x total magnification. Six random fields were captured for each experimental condition at 0, 24, 48, and 72 h after treatment. Images were analyzed using the FracLac plugin for ImageJ [31,32].

The following morphometric parameters were evaluated:

- Circularity of the convex hull (for a circle, circularity is 1).
- The perimeter in pixels of the convex hull.
- Aspect: The ratio of the major to minor axes of the convex hull.
- XY box: The ratio of width and height in pixels of the bounding box.

### Protein extraction and western blotting

The expression of EMT-marker proteins was determined by Western blot analysis. After treatments, cells were homogenized in RIPA buffer (50 mM Tris-HCl [pH 7.5], 150 mM NaCl, 1% Triton X-10, and 0.01% SDS) supplemented with a protease inhibitor cocktail (Sigma Aldrich, USA). Protein extracts were obtained by centrifugation at 12,500 rpm for 10 min at 4°C and quantified using a NanoDrop-2000 spectrophotometer (Thermo Scientific, USA). 50 μg of total protein were loaded onto a 10% polyacrylamide gel under denaturing conditions for the separation of SOX-2 and vimentin. Proteins were transferred onto nitrocellulose membranes using a semi-dry transfer system at 25 V for 45 min. Blocking was completed with 5% bovine serum albumin (BSA) for 2 h at 37°C. Membranes were incubated at 4°C for 24 h with primary antibodies against SOX2 (1:500 dilution, 39 kDa, sc-365964, Santa Cruz Biotechnology, USA) and vimentin (1:500 dilution, 57 kDa, sc-6260, Santa Cruz Biotechnology, USA). α-tubulin (1:500 dilution, at 4°C for 48 h; 50 kDa, sc-398103, Santa Cruz Biotechnology, USA) was used as a loading control. Later, membranes were incubated with the secondary antibody anti-mouse IgGMκ (1:10,000 dilution; sc-516102, Santa Cruz Biotechnology, USA) while shaking at room temperature (RT) for 1.5 h. The secondary antibody was removed and washed, and finally, the signal was detected with SuperSignal West Femto Maximum Sensitivity Substrate (Thermo Scientific, USA). Besides, 50 μg of total protein were loaded onto a 7.5% polyacrylamide gel under denaturing conditions for the separation of N-cadherin and ZO-1. Proteins were transferred to a nitrocellulose membrane under wet conditions at 250 mA for 16 h. Blocking was completed with 5% BSA at 37°C for 2 h. Membranes were incubated with primary antibodies against N-cadherin (1:300 dilution, 100 kDa, sc-8424, Santa Cruz Biotechnology, USA) at 4°C for 48 h and ZO-1 (1:250 dilution, 220 kDa, sc-33725, Santa Cruz Biotechnology, USA) at 4°C for 24 h. Later, the membranes were incubated with anti-mouse IgGMκ (1:10,000 dilution; sc-516102, Santa Cruz Biotechnology, USA) and anti-rat (1:20,000 dilution; ab95057, abcam) secondary antibodies while shaking at room temperature (RT) for 1.5 h. As a loading control, we used α-tubulin as described above.

### Statistical analysis

Data were analyzed using one-way or two-way ANOVA followed by a Tukey or Bonferroni *post hoc* test, as appropriate. GraphPad Prism 5.0 software was used for statistical analyses. A 95% confidence interval was used in all tests, and *p* < .05 was considered statistically significant. Analysis of the eta squared (η^2^) and partial eta squared (η^2^p) values were calculated as a measure of effect size considering the sources of variation: interaction, treatment, and time [33,34]. Results are expressed as mean ± SEM (Standard Error of the Mean).  The number of independent experiments (n) performed for each assay is indicated in the corresponding figure legend.

## Results

### GPER and EMT-related markers are differentially expressed among GB subtypes

**Figure 1.**
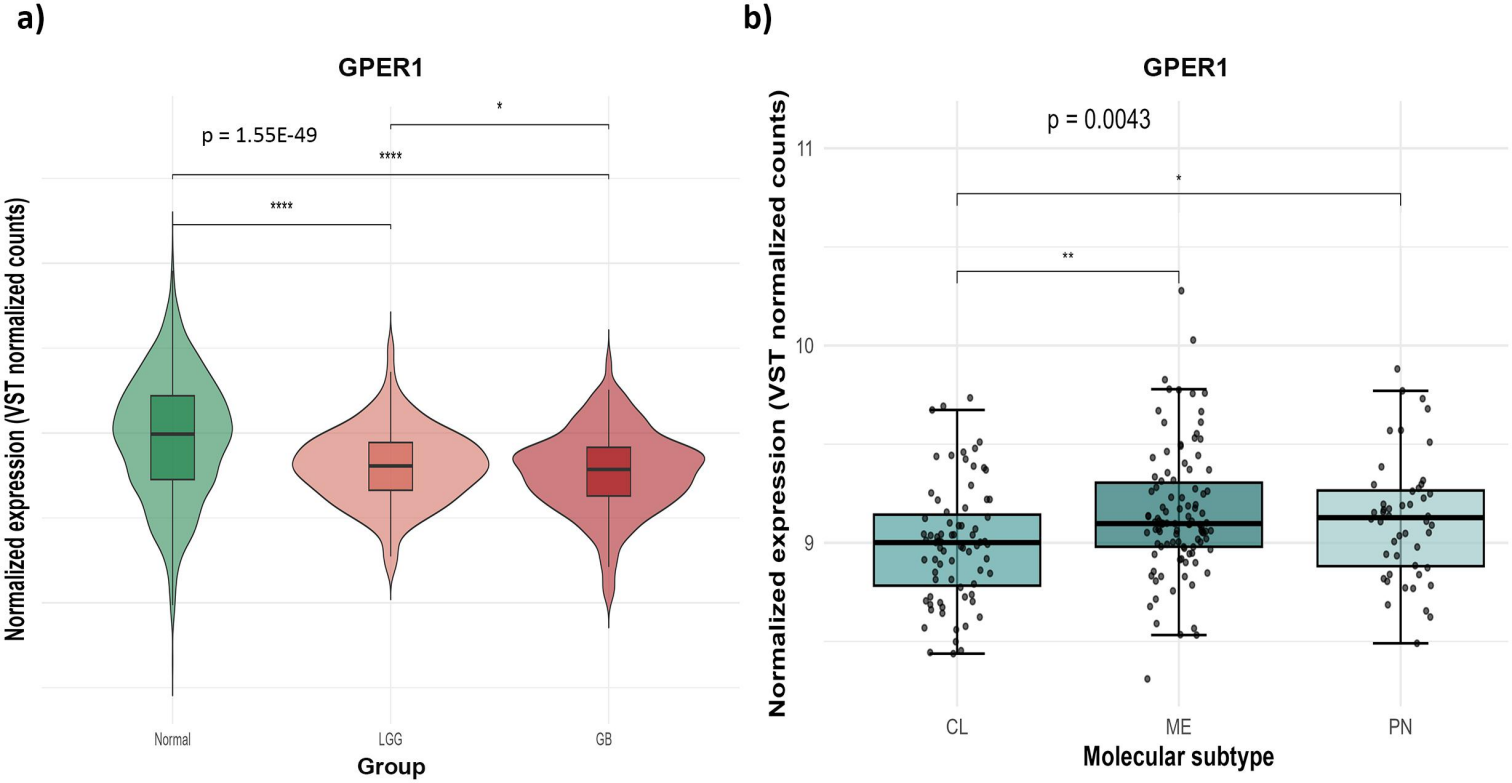
Differential expression of GPER1 in gliomas. Expression was analyzed in glioma and normal brain samples. a) GPER1 expression pattern analyzed in GB subtypes using the TCGA-GB dataset, ****p < .0001 LGG and GB vs normal. b) GPER1 expression pattern analyzed in GB molecular subtypes using the TCGA-GB dataset, *p < .05 CL vs PN, **p < .01 CL vs ME. LGG = low-grade glioma; GB = glioblastoma; CL = classic; ME = mesenchymal; PN = proneural. Statistical difference: *p < .05, **p < .01, ***p < .001, ****p < .0001, log2 Fold change >1.

**Figure 2.**
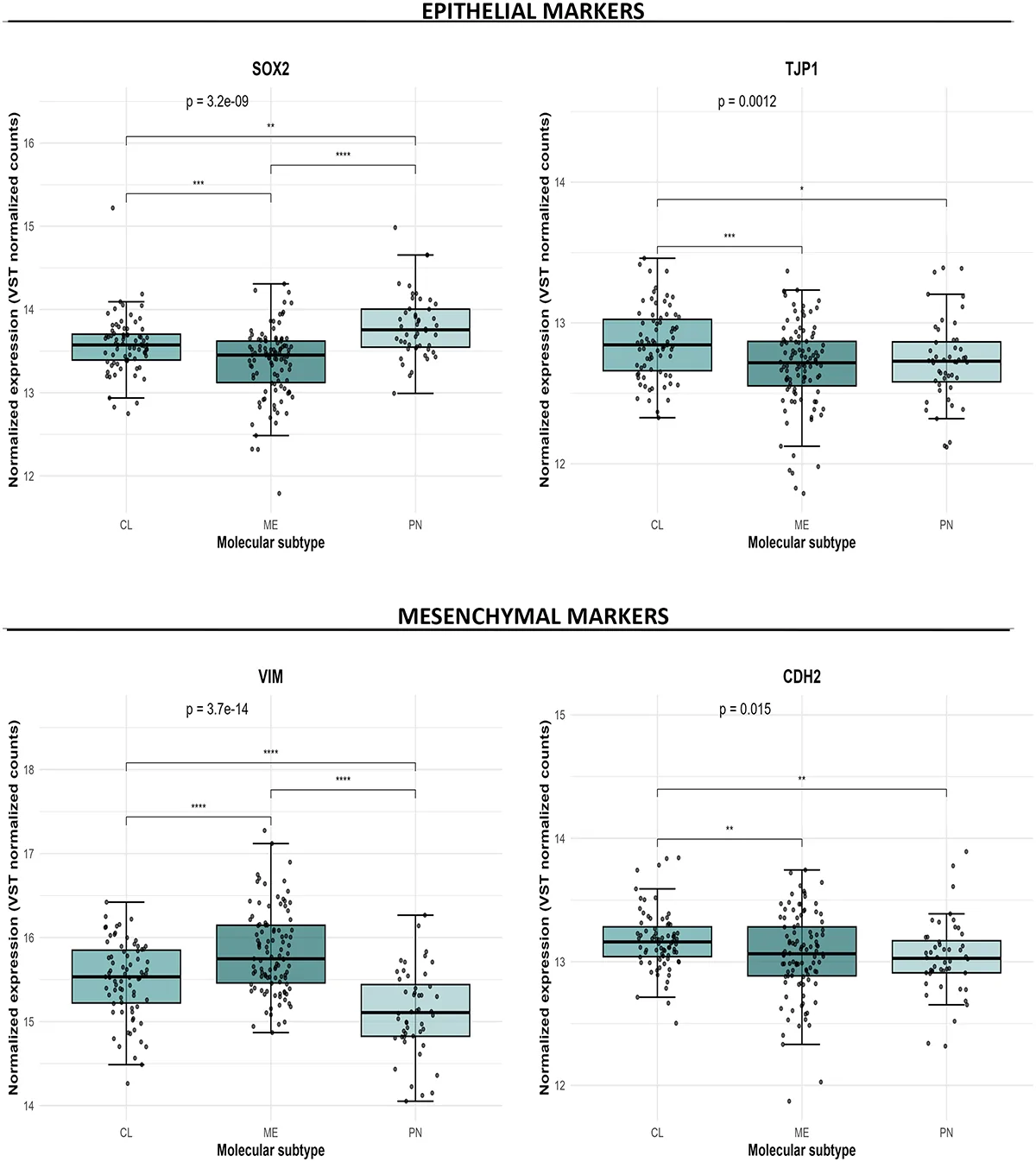
Differential expression of epithelial and mesenchymal markers in GB molecular subtypes. The TCGA-GBM dataset was used for the analysis of SOX-2: **p < .01 Cl vs PN; ***p < .01 CL vs ME; ****p < .0001 PN vs ME. TJP1: *p < .05 CL vs PN; ***p < .01 CL vs ME. Vim: ****p < .0001 ME vs. CL, PN, and CL vs. PN. CDH2: **p < .01 CL vs. ME and PN. TJP1 = ZO-1, CDH2 = N-Cadherin, CL= Classic, ME= Mesenchymal, PN= Proneural. Statistical difference: *p < .05, **p < .01, ***p < .001, ****p < .0001, log2Fold change >1.

We performed a bioinformatic analysis of RNA-Seq data from the TCGA and GTex databases, which showed decreased GPER expression in low-grade glioma and GB tissues compared with non-tumor brain samples (Figure 1a). Analysis of TCGA-GB data according to molecular subtype, proneural (PN), classic (CL), and mesenchymal (ME), revealed differential GPER expression across subtypes, with the ME and PN subtypes showing the highest levels of expression (Figure 1b). In addition, the expression of EMT-related markers was evaluated: SOX2, the TJP1 gene encoding ZO-1, VIM, and the CDH2 gene encoding N-cadherin (Figure 2). Higher expression levels of these epithelial markers were observed in the CL and PN subtypes (*p* < .001), whereas the mesenchymal marker VIM showed a higher expression in the ME subtype. Notably, the highest expression of CDH2 (N-cadherin) was observed in the CL subtype.

### GPER modulates cell growth in GB cells

As a first approach to evaluate the role of GPER in the growth of GB cells, we standardized the concentrations of the pharmacological treatments by performing cell viability assays to determine the effects of E2, the GPER agonist G-1 (10 and 100 nM), and the GPER antagonist G-15 (1, 5, and 10 μM) in the U251 and U87 human GB cell lines. Our results demonstrate that E2 and G-1 did not significantly modify viability in either cell line (Supplementary Figure 1). In contrast, treatment with G-15 (1 μM) significantly reduced cell viability in both U251 and U87 cells.

**Figure 3.**
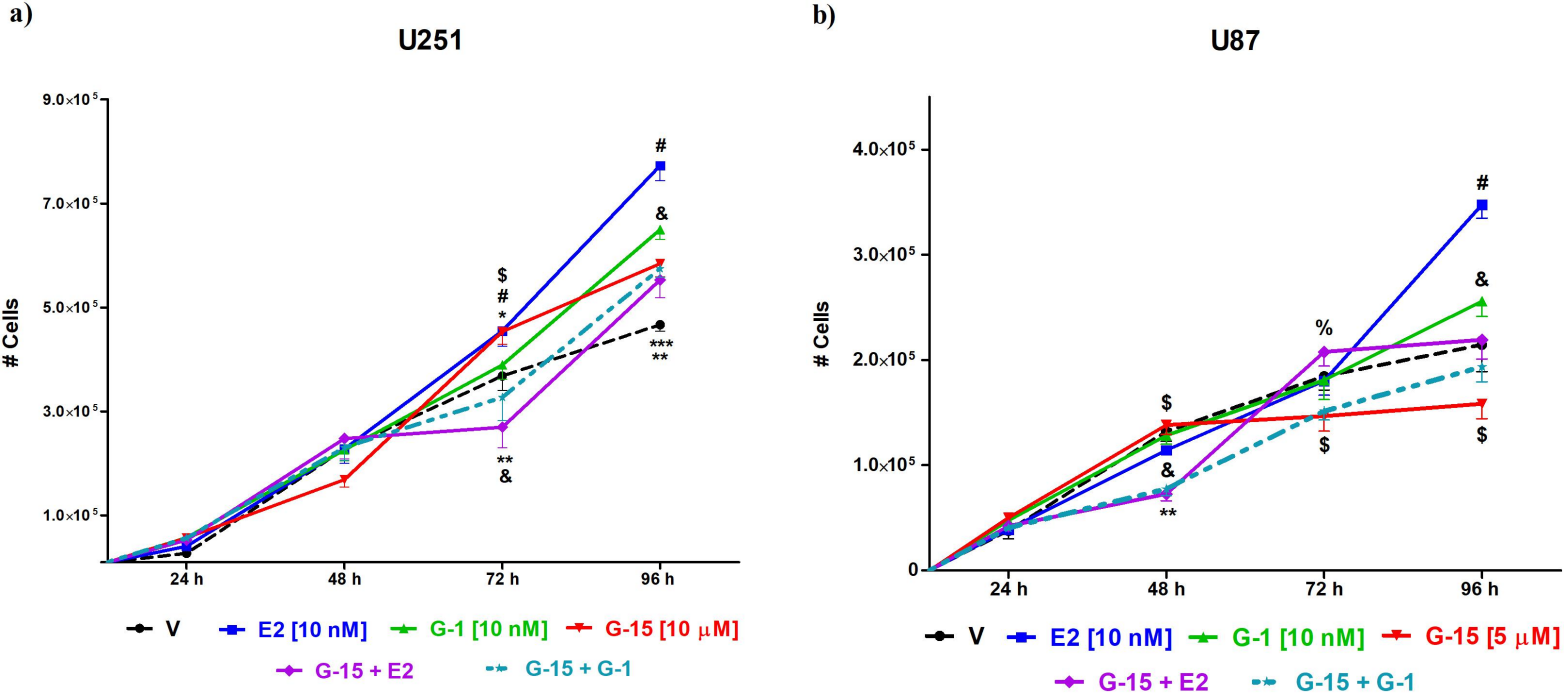
Effect of E2, G-1, and G-15 on U251 and U87 cell number. Panels a) U251 and b) U87 show cell numbers after treatment with E2, G-1, G-15, G-15+E2, G-15+G-1, and vehicle (V = CDX 0.01% + DMSO 0.01%). A two-way ANOVA with Bonferroni post hoc test were performed. Data are expressed as mean ± SEM (*n* = 4). Detailed statistics are in Supplementary tables S1-2.

Next, we studied the effects of E2, G-1, and G-15 on the number of U251 and U87 cells, using trypan blue staining after up to 96 h of treatment (Figure 3). We found that E2 and G1 significantly increased the number of cells compared with non-treated cells in the U251 line at 96 h. Interestingly, E2 treatment induced the greatest increase in cell number between 72 and 96 h. In contrast, the combined treatments G-15+E2 and G-15+G-1 significantly reduced cell number compared with E2 or G-1 alone at 72 h, suggesting that GPER blockade reverses the effects of E2 and G-1. E2 treatment significantly increased the cell number in the U87 cell line compared with the other treatment groups at 96 h. Conversely, G-15 treatment significantly reduced cell number in the U87 cell line compared with the other treatments at 96 h. The combined treatments of G-15+E2 and G-15+G-1 significantly decreased cell number compared with vehicle and G-1 treatments at 48 h.

### GPER modulates cell migration and invasion in GB cells

**Figure 4.**
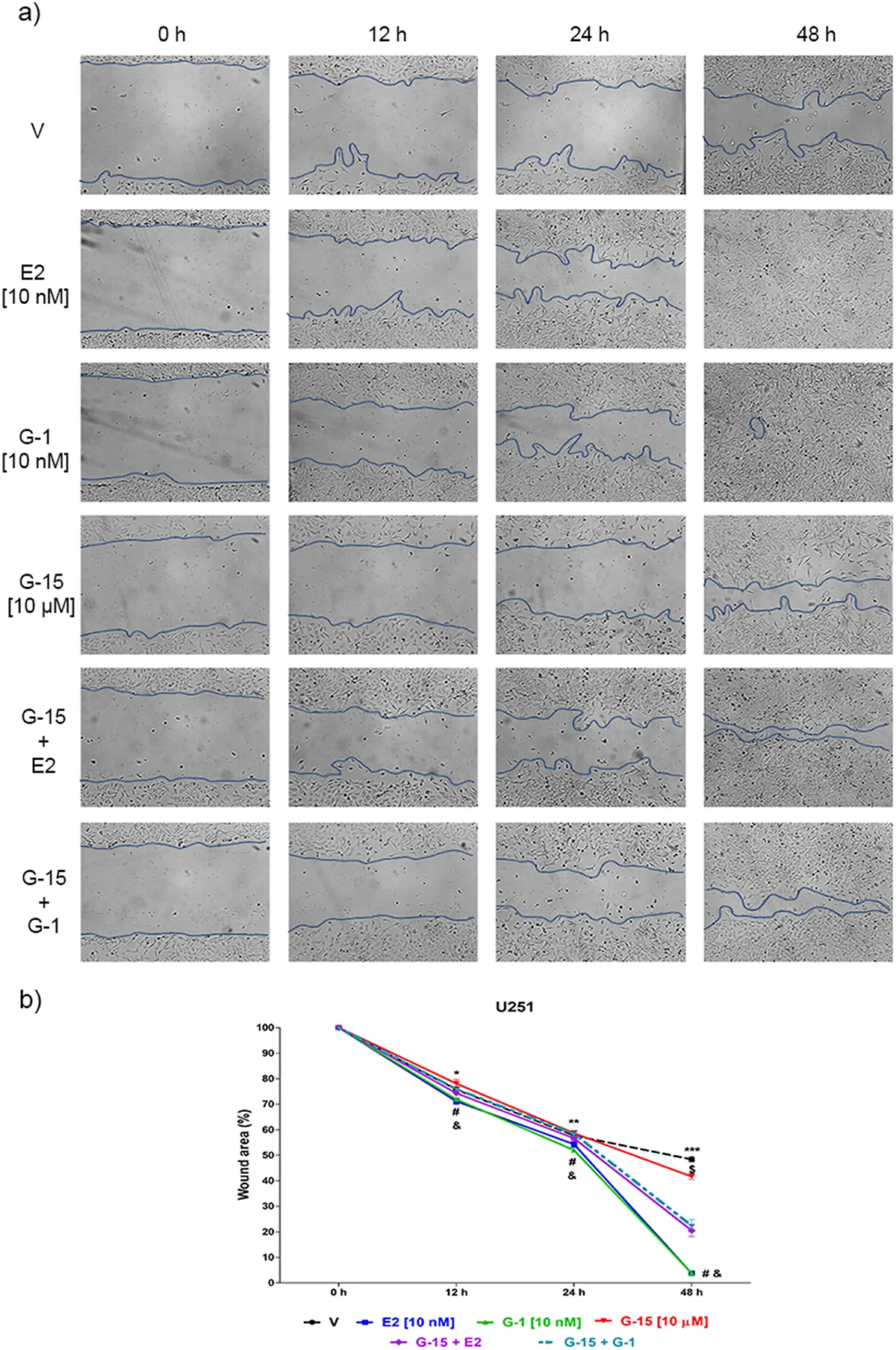
GPER activation increases cell migration in U251 cells. a) Representative images of wound closure are shown at 0, 12, 24, and 48 h after treatment with vehicle (V), E2 [10 nM], G-1 [10 nM], G-15 [10 µM], and combined treatments G-15 [10 µM] + E2 [10 nM] and G-15 [10 µM] + G-1 [10 nM]. Cells were pretreated for 1 h with cytosine β-D-arabinofuranoside hydrochloride (Ara-C, 10 µM) to inhibit DNA synthesis and minimize the contribution of cell proliferation to wound closure. b) Quantification of wound closure at the evaluated time points is shown. Statistically significant differences appear in Supplementary tables S3-4. Results were analyzed by a two-way ANOVA test followed by a Bonferroni post hoc test. Data are expressed as mean ± SEM (*n* = 5).

**Figure 5.**
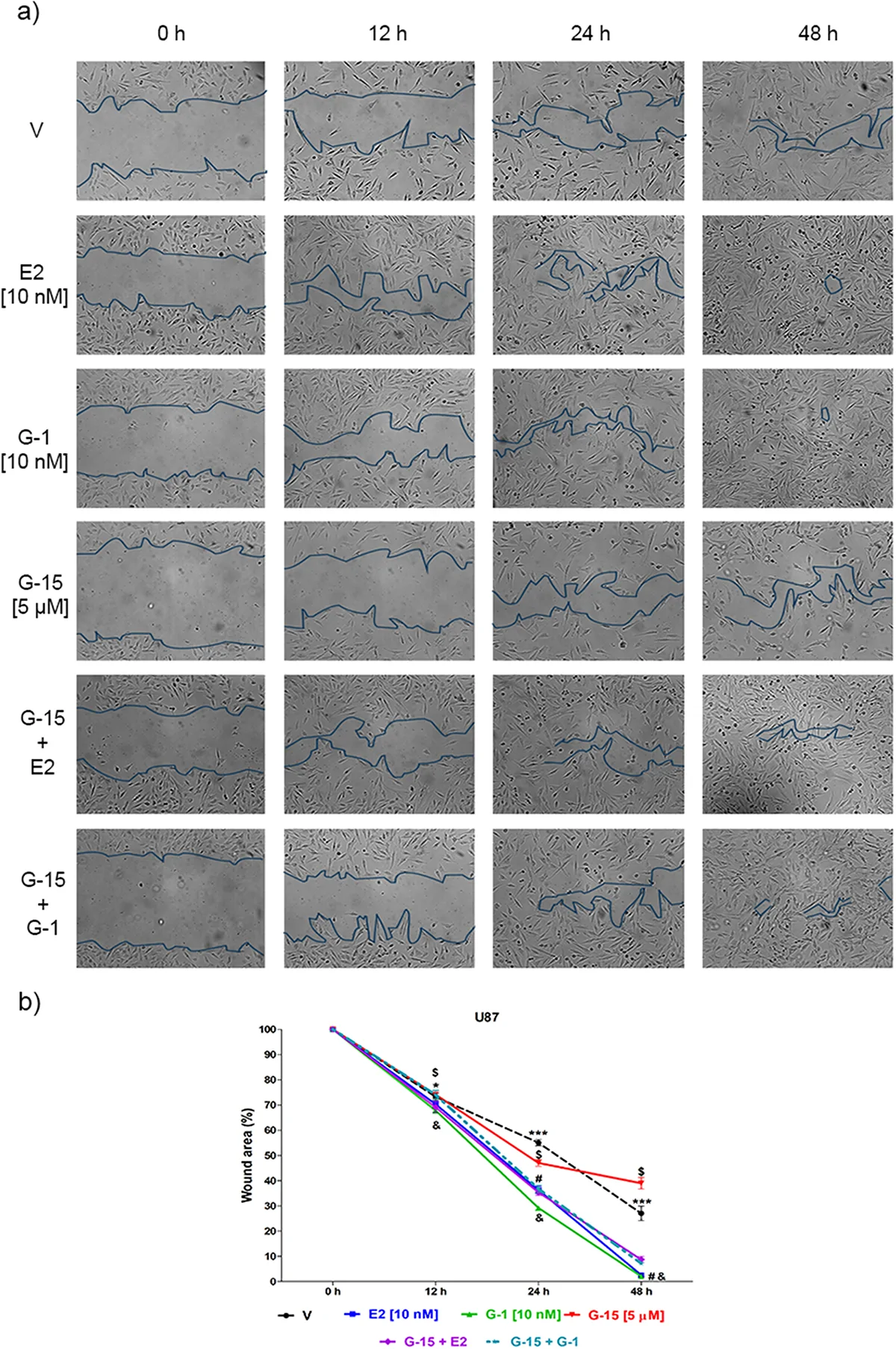
GPER regulates cell migration in U87 cells. a) Representative images of wound closure at 0, 12, 24, and 48 h for treatments with vehicle (V), E2 [10 nM], G-1 [10 nM], G-15 [5 µM], and the combined treatments G-15 [5 µM] + E2 [10 nM] and G-15 [5 µM] + G-1 [10 nM]. Cells were pretreated for 1 h with cytosine β-D-arabinofuranoside hydrochloride (Ara-C, 10 µM) to inhibit DNA synthesis and minimize the contribution of cell proliferation to wound closure. b) The quantification of the wound closure at the evaluated periods is shown. Statistically significant differences are described in Supplementary tables S3-4. The results were analyzed by a two-way ANOVA test followed by a Bonferroni post hoc test; data are expressed as mean ± SEM (*n* = 5).

Changes in cell polarity and directional motility are critical features associated with EMT-related processes that promote migratory and invasive capacities [35]. Therefore, we evaluated the effects of GPER modulation on migration and invasion in U251 and U87 GB cells. To analyze the role of GPER in cell migration, cells were treated with E2, G-1 (10 and 100 nM), and G-15 (1, 5, and 10 µM) to standardize the conditions for subsequent experiments (Supplementary Figures S2-5). Then, wound healing assays were carried out in U251 and U87 cells treated with V, E2 (10 nM), G-1 (10 nM), G-15 (10 µM) for U251 cells and G-15 (5 µM) for U87 cells, or the combined treatments G-15+E2 or G-15+G-1 (Figures 4 and 5, respectively). We found a significant increase in wound closure in U251 and U87 cells treated with E2 and G-1 from 12 to 48 h compared with the vehicle (Supplementary Tables 3 and 4); in contrast, G-15 treatment decreased migration and blocked the effects of E2 and G-1. This blockade was more evident in U251 cells at 48 h; the combined treatments G-15+E2 or G15+G-1 reduced wound closure by approximately 18% compared with the individual treatments of E2 or G-1 (Figure 4). In U87 cells, the combined treatments were like the individual treatments at 12 and 24 h (Figure 5). However, after 48 h of treatment, the combined treatments reduced wound closure by 8% compared with the individual treatments of E2 or G-1.

**Figure 6.**
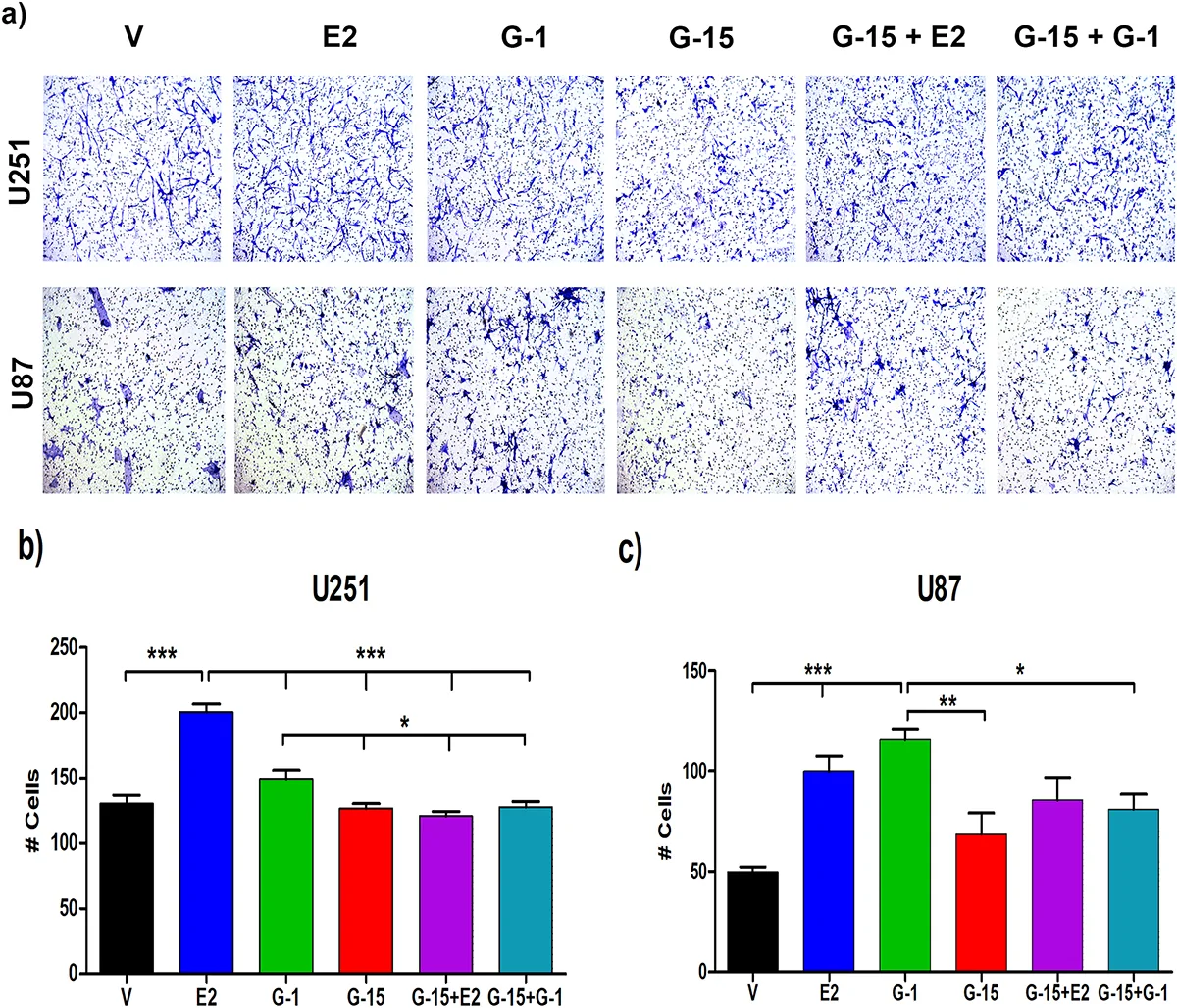
GPER activation promotes invasion in U251 and U87 cells. a) Representative images of invasion assays. b) and c) The graphs show the number of invaded cells per field for each cell line. Statistical significance for U251: ***p < .001 vs E2 and *p < .05 vs G-1; for U87: ***p < .001 vs vehicle (V) and **p < .05 vs G-1. Statistically significant differences are described in Supplementary table S5. Results were analyzed with one-way ANOVA followed by Tukey’s post hoc test. Data are presented as mean ± SEM. (*n* = 4).

The invasive capacity of GB cells was assessed using modified Boyden chamber assays. As shown in Figure 6, treatment with E2 or G-1 significantly promoted invasion in U251 and U87 cells compared with the vehicle. Cells treated with G-1 behaved similarly to those treated with E2, although E2 exhibited higher effects than G-1 (Figure 6a). A similar pattern was observed in U87 cells (Figure 6b); however, G-1-treated U87 cells showed a more pronounced invasive effect than G-1-treated U251 cells.

Conversely, treatment with the GPER antagonist G-15 produced effects opposite to those observed with E2 and G-1, suggesting that GPER contributes to the acquisition of migratory and invasive properties associated with EMT-like processes in GB cells. Consistent with these results, cells treated with G-15+E2 or G-15+G-1 exhibited a similar behavior to that of cells treated with G-15 alone. Taken together, these findings suggest that pharmacological inhibition of GPER reduces the invasive capacity of U251 and U87 cells.

### GPER regulates EMT-related processes and the expression of EMT-related markers in GB cells

**Figure 7.**
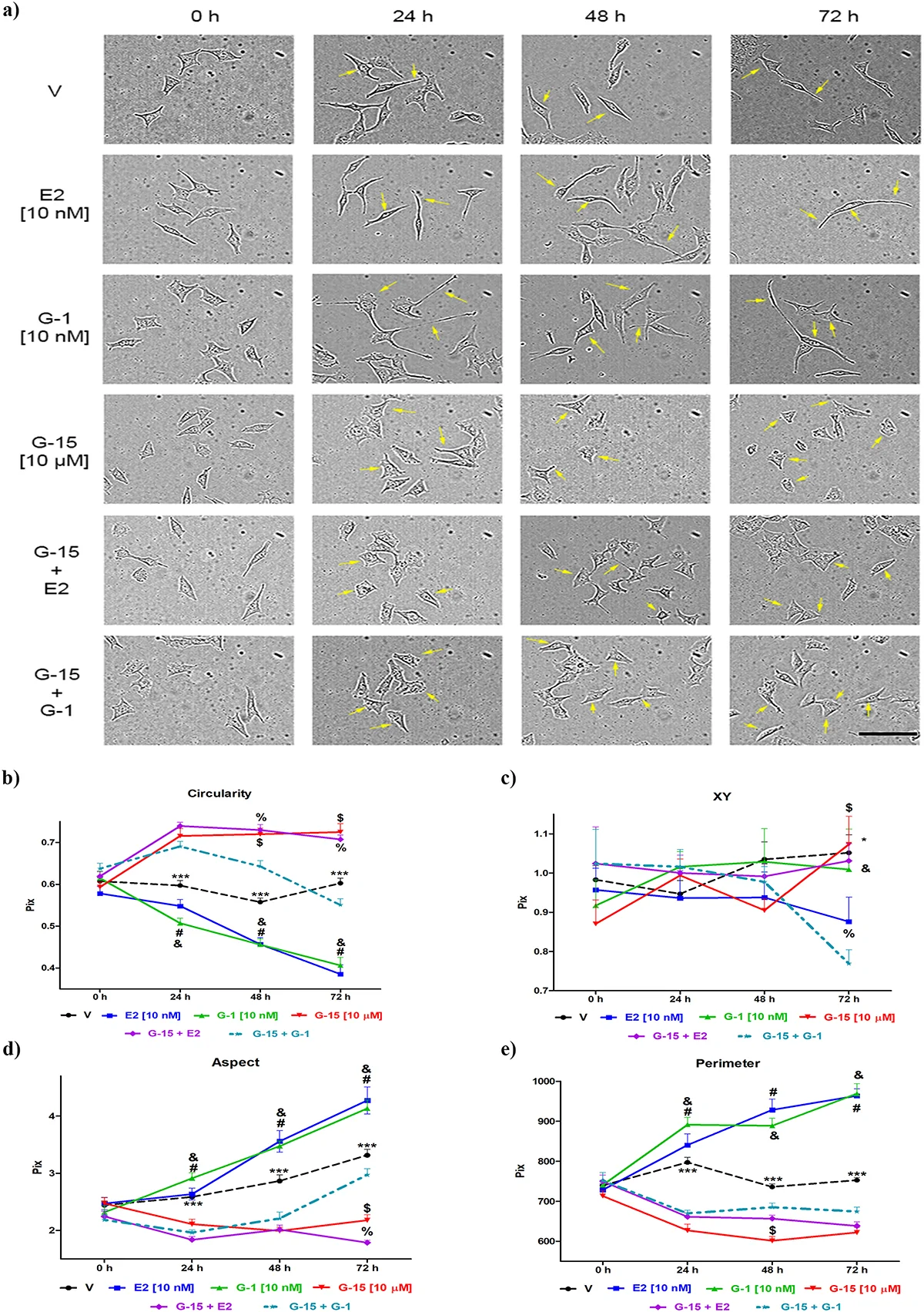
Changes in morphology of U251 cells induced by GPER activation. a) Shows representative images of cell morphology changes indicated by yellow arrows, at 24, 48, and 72 h for treatments with V (CDX 0.01% + DMSO 0.01%), E2 [10 nM], G-1 [10 nM], G-15 [10 µM], and the combined treatments G-15+E2 and G-15+G-1. The bottom part shows graphs for the geometric parameters evaluated during the trial: b) Circularity, c) XY box, d) Aspect, e) Perimeter. Statistically significant differences are described in Supplementary tables S6-7. Black scale bar = 100 µM, Pix = Pixels. Results were analyzed using a two-way ANOVA test, followed by a Bonferroni post hoc test. Data are expressed as mean ± SEM (*n* = 4).

**Figure 8.**
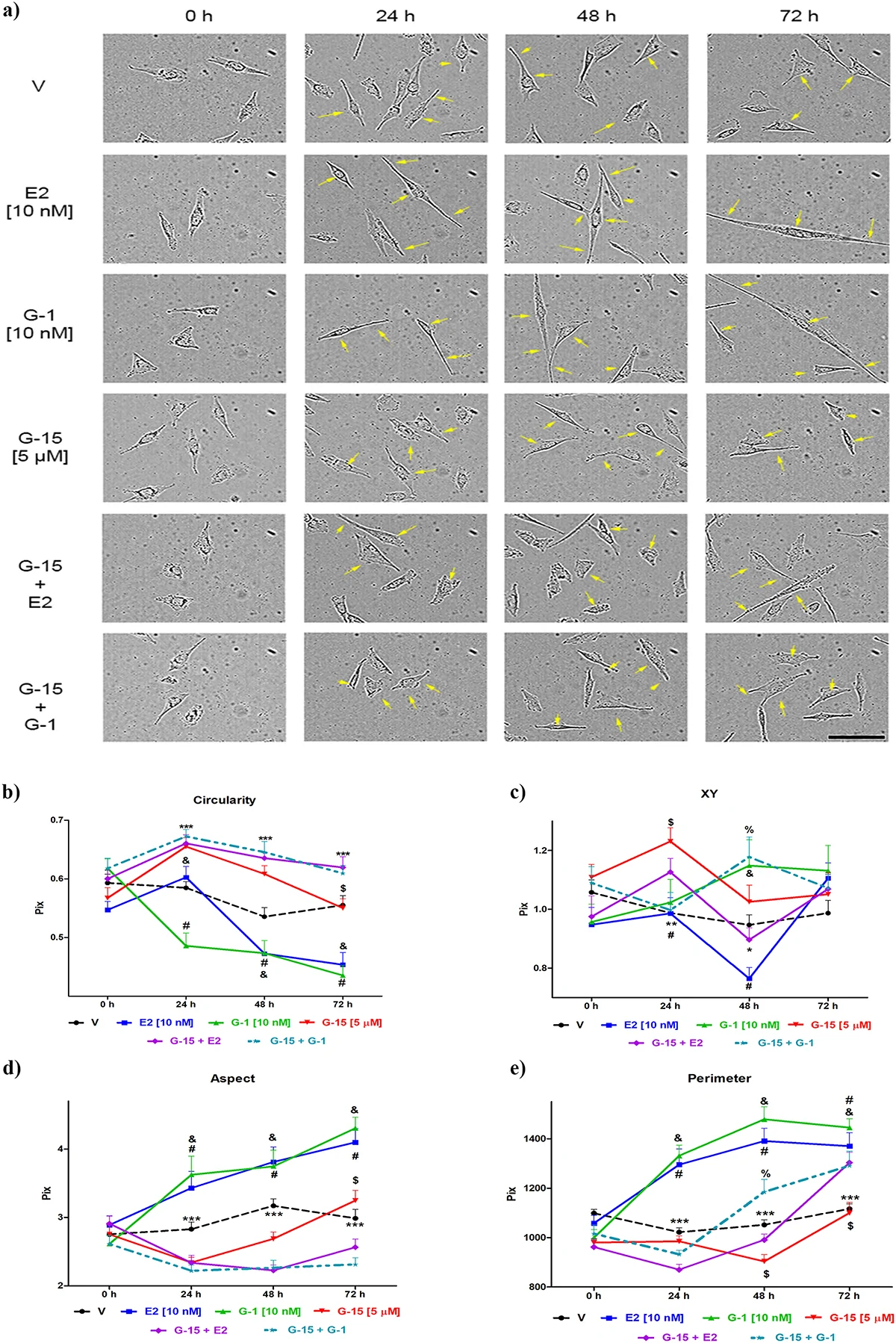
GPER activation modifies morphology in U87 cells. a) Shows representative images of cell morphology changes, indicated by yellow arrows, at 24, 48, and 72 h for treatments with V (CDX 0.01% + DMSO 0.01%), E2 [10 nM], G-1 [10 nM], G-15 [5 µM], and the combined treatments G-15+E2 and G-15+G-1. The bottom part shows graphs for the geometric parameters evaluated during the trial: b) Circularity, c) XY box, d) Aspect, e) Perimeter. Statistically significant differences are described in Supplementary tables S8-9. Black scale bar = 100 µM, Pix= pixels. Results were analyzed using a two-way ANOVA test followed by a Bonferroni post hoc test. Data are expressed as mean ± SEM (*n* = 4).

Changes in cell morphology are characteristic features associated with EMT-related processes and are driven by cytoskeletal reorganization to acquire an invasive phenotype [36]. We found that in U251 and U87 cell lines (Figures 7 and 8, respectively), geometric parameters of cells shifted from a polygonal shape associated with an epithelial phenotype to a fusiform shape associated with a mesenchymal-like phenotype. In cells treated with E2 and G-1, circularity and XY box parameters decreased, while aspect ratio and perimeter were significantly increased compared with the vehicle. These changes were detected from 24 to 72 h after treatment in most of the evaluated parameters. The antagonist G-15 treatment showed an effect opposite to that observed with E2 and G-1 treatments. The circularity and XY box of the cells increased, while the perimeter significantly decreased. Notably, cells treated with G-15+E2 or G-15+G-1 exhibited a morphology comparable to that of cells treated with G-15 alone. Together, these findings indicate that pharmacological inhibition of GPER attenuates mesenchymal-like morphological changes in GB cells.

**Figure 9.**
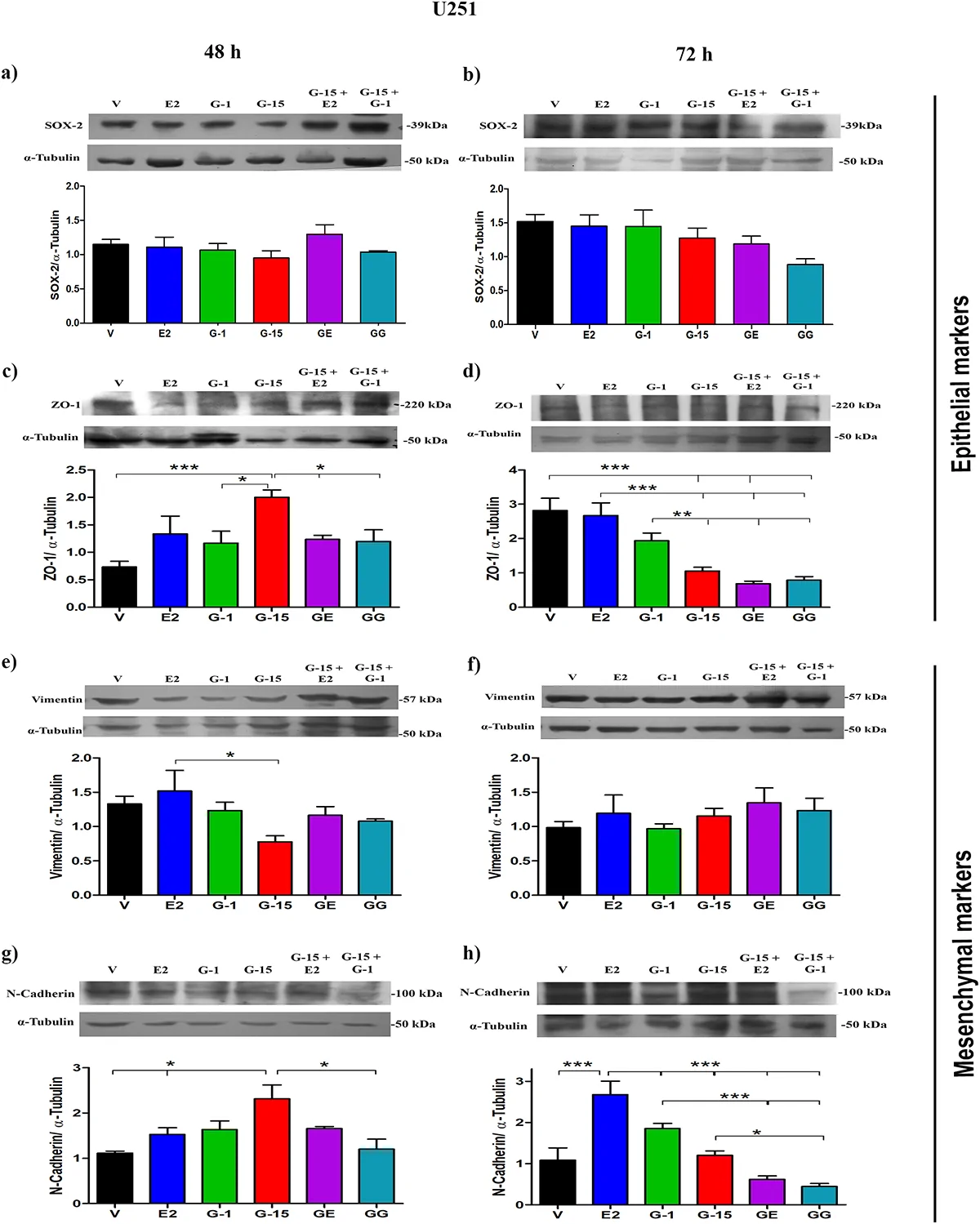
Effects of GPER activation in the expression of SOX-2, ZO-1, vimentin, and N-cadherin markers in the U251 cell line. The upper panel shows representative Western blots for SOX-2 (39 kDa), ZO-1 (220 kDa), vimentin (57 kDa), and N-cadherin (100 kDa), and the lower panel shows the respective densitometric analyses for the U251 cell line. a), c), e), g) the analysis is shown after 48 h treatments c) ***p = 6.95 × 10^−6^ and *p = .015 vs G-15, e) *p = .018 vs E2, g) *p = .024 vs G-15. b, d, f, h) the analysis is shown after 72 h treatments. d) ***p = 2.1 × 10^−8^ vs. V or *p* = 1.1 × 10^−8^ E2 and **p = .0011 vs. G-1, f) no statistical difference was found in vimentin expression, and h) ***p = 1.5 × 10^−6^ vs. E2, ***p = 1.6 × 10^−5^ vs. G-1, as well as *p = .032 vs. G-15. The results obtained were analyzed using a one-way ANOVA test followed by a Tukey post hoc test. Data are expressed as mean ± SEM (*n* = 4). GE: G-15+E2, GG: G-15+G-1.

To further characterize EMT-like phenotypic changes, we evaluated the expression of ZO-1 as a junction-associated protein, SOX2 as a stemness-associated factor, and the mesenchymal markers vimentin and N-cadherin. As shown in Figure 9, no statistical difference was found in the expression of SOX-2 in the U251 cell line under any of the treatment conditions (Figure 9a,b). In U251 cells, ZO-1 expression was significantly increased after G-15 treatment at 48 h (*p* < .001). However, at 72 h, ZO-1 expression was decreased in cells treated with G-15, as well as in the combined treatments G-15+E2 and G-15+G-1, compared with the vehicle (Figure 9c,d).

**Figure 10.**
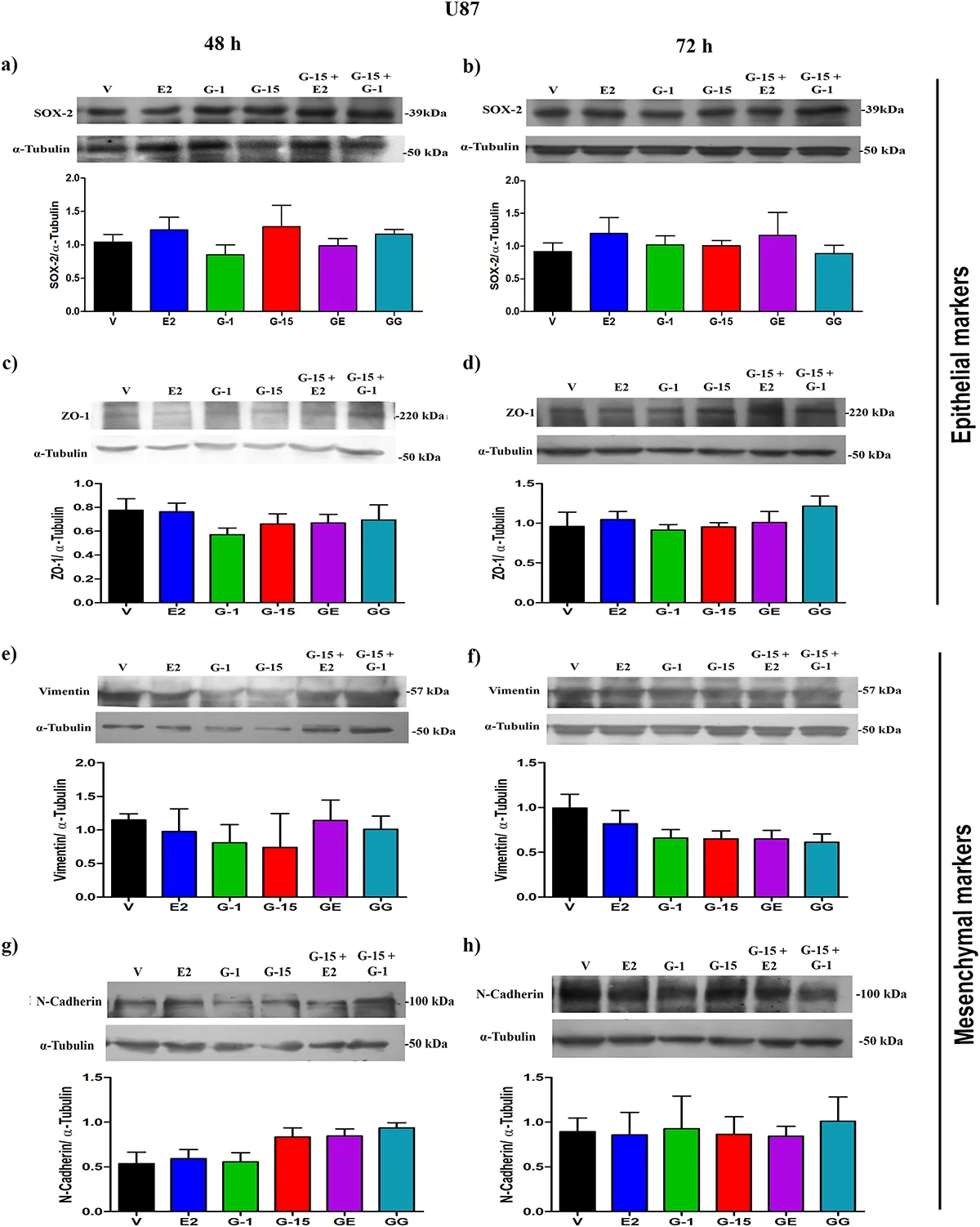
Effects of GPER activation on the expression of SOX-2, ZO-1, vimentin, and N-cadherin markers in the U87 cell line. The upper part shows representative Western blots for SOX-2 (39 kDa), ZO-1 (220 kDa), vimentin (57 kDa), and N-cadherin (100 kDa), and the lower part shows the respective densitometric analyses for the U87 cell line. a), c), e), and g): the analysis is shown after 48 h of treatment. b), d), f), and h): the analysis is shown after 72 h of treatment. The results obtained were analyzed using a one-way ANOVA test followed by a Tukey post hoc test. Data are expressed as mean ± SEM (*n* = 4). GE: G-15+E2, GG: G-15+G-1.

Regarding mesenchymal markers, vimentin expression significantly increased after 48 h of E2 treatment (*p* < .05) compared with G-15-treated cells in the U251 cell line; however, this effect was not maintained at 72 h (Figure 9e,f). Conversely, we found that N-cadherin expression increased in U251 cells treated with G-15 during 48 h (*p* < .05). Notably, the treatments with E2 and G-1 increased N-cadherin expression at 72 h (*p* < .001 and *p* < .05), while treatment with G-15, G-15+E2, and G-15+G-1 decreased the expression of this marker compared to cells treated with E2 (Figure 9g,h). Interestingly, no statistically significant changes in marker expression were observed in the U87 cell line at either 48 or 72 h after treatments (Figure 10).

## Discussion

GB is the most aggressive tumor of the CNS, with a higher incidence in men than in women [3]. Several studies have demonstrated the impact of the endocrine environment on GB progression [37]. In this context, one sex hormone of particular interest is E2, which modulates various aspects of GB biology [16]. Tumor-associated processes such as proliferation, migration, invasion, and EMT-related phenomena have been widely linked to the roles of the intracellular receptors ERα and ERβ. In GB, GPER has recently been identified, and its role in the progression of this neoplasm is unclear. The present study provides pharmacological evidence supporting the involvement of GPER activation in EMT-related processes, including cell migration and invasion, in human GB-derived cells.

A hypothesis has been proposed that GPER expression is inversely proportional to tumor malignancy, a notion supported by our *in silico* analysis, which showed decreased GPER expression in low-grade glioma and GB tissues compared to non-tumor samples [15,38–40]. Interestingly, an analysis using the TCGA-GBM database revealed that among women patients who underwent chemoradiotherapy, those with high GPER expression had favorable overall survival; however, this association was not observed in male patients [21]. These findings suggest that the prognostic significance of GPER is likely influenced by patient sex, treatment, and tumor molecular context rather than by GPER expression alone. Therefore, further studies are required to clarify the clinical relevance of GPER as a prognostic biomarker in GB.

In addition, our previous study demonstrated that E2 modulates GPER expression after 24 h of treatment in GB-derived cells [22]. However, GPER expression was not evaluated after prolonged E2 exposure in the present study. Therefore, it remains unknown whether sustained E2 treatment further alters GPER expression or contributes to persistent activation of downstream signaling pathways associated with EMT-related phenotypic changes. Future studies addressing the temporal regulation of GPER expression and its downstream signaling will be important for better understanding the role of GPER in GB progression.

We then analyzed GPER expression across GB molecular subtypes, and we found higher expression in the PN and ME subtypes. It should be noted that previous studies have determined that the U251 cell line expresses markers associated with the CL/PN molecular subtype. In contrast, the U87 cell line expresses markers of the PN/ME molecular subtype (both cell lines are derived from male patients) [5,41]. This is consistent with experimental evidence of differential GPER expression in cell lines and GB samples from the TCGA.

It has been reported that E2 through ERα induces cell growth in the U373 and D54 lines derived from human astrocytomas [17]. This is consistent with our findings, which showed that treatment with E2 and G-1 increased cell numbers in human GB-derived cell lines. On the other hand, reports of combined treatments with G-15+E2 and G-15+G-1 indicate a blockade of GPER function [26,42,43]. We evaluated these treatments in our study model and found a decrease in cell numbers at 72 h in U251 and at 48 h in U87 cells compared with E2 and G-1 treatments. These differential responses may be associated with variations in GPER expression levels, which are reportedly higher in U87 cells. This is consistent with reports in breast cancer models, where GPER expression has been associated with changes in cell viability, proliferation, motility, migration, and invasion [44,45].

The acquisition of migratory and invasive properties through EMT-related processes is considered a key event in tumor progression. Our findings show that the migratory capacity of GB cells increases with E2 and G-1 treatments, whereas G-15 inhibits it, highlighting the role of GPER in GB migration as reported in other cancer types [43,46–48]. The combined treatment of G-15+E2 and G-15+G-1 decreased migratory capacity compared to individual treatments with E2 and G-1, as observed in ovarian and breast cancer cell models [26,42].

GPER has been shown to regulate migration through Rho GTPases in various cellular models by activating the PI3K/FAK/Akt/Rho/Rac signaling axis [42,49], as GTPases of the Rho family are known to be central regulators of cell migration, acting as molecular switches that associate with lipid membranes and maintain migration [36]. GPER also promotes F-actin cytoskeletal reorganization via the PLCβ/PKC and Rho/Rac/LIMK/TAZ pathways [50]. Moreover, GPER activity has been linked to proliferation, migration, and invasion through the miR-124/CD151 pathway [26]. Although these signaling pathways provide plausible mechanisms underlying the effects observed in the present study, they were not directly evaluated in our experimental model. Accordingly, future studies will be required to determine whether signaling pathways such as PI3K/FAK/Akt/Rho/Rac, PLCβ/PKC, and the miR-124/CD151 signaling axis mediate GPER’s effects on EMT-related phenotypic changes, migration, and invasion in GB cells.

Our results indicate that GPER promotes cell invasion when stimulated by its agonists, E2 or G-1, whereas combined treatments with G-15+E2 or G-15+G-1 inhibited this invasive capability. However, a striking difference is observed in the U87 cell line, where treatment with G-1 significantly increases invasive capacity, a finding directly linked to receptor expression. Research on ovarian cancer has shown that GPER promotes migration and invasion by increasing MMP-9 expression [51,52]. Besides, it has been demonstrated that GPER promotes PI3K/AKT/MMP-9 signaling in renal cancer cells [53]. This background suggests that GPER may contribute to GB invasiveness through signaling pathways regulating MMP-9, although this possibility warrants further investigation.

EMT-related processes are among the mechanisms that generate different cellular phenotypes. These phenotypes play a fundamental role in GB’s invasive capabilities [9–11]. The epithelium consists of one or more layers of differentiated cells that attach to the basement membrane through narrow spaces, hemidesmosomes, and adherents junctions, exhibiting a static apical-basal polarity. However, when cells transition to a mesenchymal state, cell-cell connections break, promoting the reorganization of the basement membrane and cytoskeleton. This process is reversible and is known as MET [13]. In this regard, the factors that induce EMT in other cancers can also activate mesenchymal characteristics in gliomas. The key invasive mechanisms overlap between CNS neoplasms despite lacking this critical epithelial tissue component [14].

We conducted an assessment to address the role of GPER in EMT-related morphological changes. Treatment with E2 promoted the acquisition of mesenchymal-like phenotype, as previously reported by our group [15,54]. G-1 also promoted this mesenchymal morphological change, indicating an active role for GPER in regulating EMT-related processes in GB cells. In contrast, cells treated with G-15 exhibited morphological characteristics associated with a more epithelial-like phenotype. It is important to note that in several cancers, the EMT-MET balance typically does not reach completion, resulting in a hybrid epithelial/mesenchymal phenotype that contributes to so-called ‘epigenetic heterogeneity’ [13]. Our results suggest that GB cells can exhibit this hybrid phenotype and that GPER activation can modulate their transition between these phenotypic states. The effects observed in the combined treatments of G-15+E2 and G-15+G-1 are similar to those observed with the individual treatment with G-15.

Molecular characterization has served as the basis for classifying GB into three molecular subtypes: PN, CL, and ME [7,55,56]. The ME subtype is the most aggressive one [8,56], and a change from the PN subtype to the ME subtype, called Proneural-Mesenchymal Transition (PMT), has been described. These molecular transition processes are similar to those that drive EMT in cells from other cancers [57–59].

During tumor formation, certain epithelial markers such as E-cadherin, claudins, ZO-1, and cytokeratins, which are important in maintaining the epithelial phenotype, decrease their expression. In contrast, mesenchymal markers such as N-cadherin, fibronectin, and Vimentin are upregulated, leading to this shift in the mesenchymal phenotype [60]. Therefore, in the present study, we evaluated different EMT markers that, in our GB model, would properly describe a PMT.

It should be noted that EMT is an important inducer of the stem cell phenotype by conferring certain characteristics of cellular plasticity. In GB, it has been reported that the ME subtype usually expresses stem cell markers [13,14]. Among these, SOX-2 is particularly notable for its role in stemness and maintenance of self-renewal. Keller and colleagues have linked SOX-2 function to modulation of EMT-related processes in lines derived from human GBs, as SOX2 expression is lost during differentiation of a PN to an ME subtype [61]. A study led by Berezovsky determined that SOX-2 is part of the genetic signature of the PN and CL molecular subtypes, consistent with our *in silico* analysis. Furthermore, the authors reported that SOX-2 expression did not show significant differences at either the protein or transcriptional level in GB samples [62]. Interestingly, we also found no differences in SOX-2 expression after E2, G-1, and G-15 treatments, suggesting that GPER does not play a role in the mechanisms of gene amplification, promoter hypomethylation, and activation of signaling pathways that regulate SOX-2 expression in GB [62,63].

In the U251 cell line, which has been linked to the CL/PN subtype, the ZO-1 marker expression was increased following G-15 treatment at 48 h, suggesting that GPER blockade promotes an epithelial phenotype. Controversially, we observed that ZO-1 expression decreased at 72 h of G-15 treatment. A similar effect was observed in a murine model, in which the antagonist G-15 (0.3 mg/kg) decreased ZO-1 expression in crypt cells [64]. Additionally, our *in silico* analysis indicated greater TJP1 (the gene encoding ZO-1) expression in the CL and PN subtypes. Our results demonstrate that GPER can play a dual role, depending on treatment timing and the underlying molecular context, as no statistically significant changes in ZO-1 expression were observed in the U87 cell line (PN/ME subtype).

Regarding mesenchymal markers, we evaluated vimentin, an intermediate filament protein involved in regulating cell mechanics and mechanosensitive transduction, motility, and healing by coordinating the EMT process [65,66]. Therefore, it is a key protein in the characterization of EMT. Our *in silico* analysis revealed elevated vimentin expression in the ME molecular subtype, consistent with previous reports [67]. Similarly, GB samples have been shown to exhibit higher vimentin expression compared to low-grade and non-tumor glioma samples, in addition to their role in GB cell invasion [68]. Moreover, our results showed increased vimentin expression in cells treated with E2, corroborating a previous report from our group [15]. However, we found no differences in vimentin expression in cells treated with G-1 or G-15, suggesting that vimentin is not a GPER target in our cell model. In contrast, evidence from cervical cancer has shown that G15 treatment activates the GPER/PI3K/AKT/PD-L1 signaling pathway and directly reduces the expression of vimentin and N-cadherin [69].

The switch involving the negative regulation of E-cadherin and the positive regulation of N-cadherin is a hallmark of EMT. However, this is a simplified model of cadherin expression, accepted only for epithelial tumors. Previous works clearly indicate that N-cadherin is expressed in 81.5% of GBs, whereas E-cadherin is expressed in only 31%, suggesting that N-cadherin is more prevalent in GBs than in low-grade gliomas [70]. Our *in silico* analysis showed that CDH2 (the gene encoding N-cadherin) was more highly expressed in the CL molecular subtype. We also found that in the U251 cell line, N-cadherin expression was increased at 48 h in G-15-treated cells, whereas at 72 h it was increased after treatment with E2 and G-1.

In contrast, treatment with G-15, G-15+E2, and G-15+G-1 decreased the expression of this marker at 72 h. This finding aligns with previous research, which reported that E2 promotes the expression of this marker through ERα [15,69]. Taken together, these data indicate that GPER regulates N-cadherin expression in the U251 cell line and suggest that GPER activation promotes a partial EMT-like phenotype.

Finally, we found no significant changes in marker expression in the U87 cell line across all markers evaluated. This cell line belongs to the PN/ME molecular subtype and exhibits higher GPER expression. Treatment with E2 and G-1 increased cell numbers, whereas G-15 reduced them. Furthermore, G-1 treatment significantly increased invasive capacity, resulting in a hybrid epithelial/mesenchymal phenotype. This reinforces the dynamic and atypical role of the receptor in response to underlying molecular stimuli and contexts.

Although the expression of SOX2, ZO-1, vimentin, and N-cadherin provides evidence of phenotypic changes associated with EMT-like processes, we recognize that this marker panel does not fully capture the complexity of mesenchymal transition programs in GB. Additional EMT-associated transcription factors, including Snail, Slug, Twist, and ZEB1/2, as well as other epithelial- and mesenchymal-associated markers, should be evaluated in future studies to further characterize GPER-mediated phenotypic plasticity.

Furthermore, although the present study provides pharmacological evidence supporting the involvement of GPER-associated signaling in EMT-related phenotypic changes, migration, and invasion of GB cells, several limitations should be acknowledged. GPER function was evaluated exclusively through pharmacological modulation using G-1 and G-15; therefore, potential off-target effects cannot be completely excluded. Future studies employing complementary genetic approaches, such as siRNA-, shRNA-, or CRISPR/Cas9-mediated GPER silencing, together with analyses of downstream signaling pathways, will be required to definitively establish the molecular mechanisms by which GPER regulates these cellular processes. Experimental approaches involving the inhibition or degradation of ERα and ERβ will also be required to delineate the individual contributions of GPER and the classical estrogen receptors to the effects observed in this study.

In addition, the present study used two well-established human GB cell lines representing distinct molecular backgrounds. Although these models provide a valuable platform for investigating GPER function, they do not fully reflect the biological heterogeneity of GB. Therefore, validation of these findings in patient-derived GB cells and other clinically relevant experimental models will be important to determine the generalizability and translational relevance of the observed effects.

## Conclusion

This study provides pharmacological evidence that activation of GPER-associated signaling in GB cells promotes a partial EMT-related phenotype by inducing cell morphological changes, increasing migratory and invasive capacities, and modulating the expression of epithelial and mesenchymal markers, in a context-dependent manner, influenced by the cell’s underlying molecular profile. However, to provide a detailed and integrative panorama of GPER’s role in GB progression, evaluating downstream signaling pathways using diverse experimental strategies is required.

## Supplementary Material

Supp Figs Pena_Gutierrez el al 2026- clean.docx

## Data Availability

The data that support the findings of this study are available from the corresponding author, [ICA], upon reasonable request.
