# Supplementary material for: G protein-coupled estrogen receptor (GPER) modulates epithelial-mesenchymal transition-like phenotype in human glioblastoma cells
Source: Cell Adh Migr. 2026 Aug 21;20(1):2720325. doi: 10.1080/19336918.2026.2720325 (PMC13502043; doi:10.1080/19336918.2026.2720325)
Supplement: Supplementary file 1 [file KCAM_A_2720325_SM0847.docx]

**Supplementary Figure 1. Effects of GPER modulators on U251 and U87 cells viability.** Cell viability is shown after treatment with different concentrations of E2, G-1, and G-15 for 24 h. Results were analyzed using a two-way ANOVA followed by the Bonferroni post hoc test; data are expressed as mean ± S.E.M (n=4). In c) statistical analysis shows significant differences in U251 cells: **p=0.0028 G-15 [5 µM] vs G-15 [10 µM], ***p=2.8x10^-6^ G-15 [1 and 5 µM] vs. V, p=2.5x10^-7^ G-15 [1 µM] vs. G-15 [10 µM]. In d) statistical analysis shows significant differences in U87 cells: **p=0.0012 G-15 [1 µM] vs. V, and p= 0.0018 G-15 [5 µM] vs G-15 [10 µM], ***p=2.4x10^-6^ G-15 [10 µM] vs. V.

**Supplementary Figure 2. E2 and G-1 increase migration in U251 cells.** In a) representative images of wound closure at 0, 12, 24, and 48 h are shown for V (CDX 0.01%), E2 10, and 100 nM treatments as well as V (DMSO 0.01%), G-1 10, and 100 nM. The bottom panels show the quantification of the wound area at the evaluated time points. In b) quantification of wound closure produced by E2 at 10 and 100 nM, and in c) by G-1 at 10 and 100 nM. In b and c ***p=2.73x10^-11^ E2 or p=3.64x10^-6^ G-1 vs. V, from 12 h-48 h. &, p=0.0000307 E2 [10 nM] vs E2 [100 nM] at 12 and 24 h, $, p=0.024 G-1 [10 nM] vs G-1 [100 nM] at 24 h and &, p=0.00046 G-1 [10 nM] vs G-1 [100 nM] at 48 h. The size effects for treatment in b) η^2^=0.13, η^2^p=0.11 and c) η^2^=0.15, η^2^p=0.13. The results were analyzed by a two-way ANOVA test followed by the Bonferroni post hoc test; data are expressed as mean ± S.E.M (n=5).

**Supplementary Figure 3. E2 and G-1 increase cell migration in the U87 cell line.** a) Representative images of wound closure at 0, 12, 24, and 48 h are shown for treatments with V, E2, or G-1 at 10 and 100 nM. In b) quantification of wound closure for E2 at 10 and 100 nM. c) quantification of wound closure for G-1 at 10 and 100 nM. In b and c ***p =0.00046 E2 or p=0.00084 G-1 vs. V, from 12-48 h. The size effects for treatment in b) η^2^=0.13, η^2^p=0.11 and c) η^2^=0.15, η^2^p=0.13. The results were analyzed by a two-way ANOVA test followed by a Bonferroni post hoc test; data are expressed as mean ± SEM (n=5).

**Supplementary Figure 4. G-15 effects on cell migration in the U251 line.** In a) Wound closure at 0, 12, 24, and 48 h, for treatments with G-15 at 1, 5, and 10 µM, and V (DMSO 0.01%). Panel b) shows the quantification of the wound area at the evaluated periods of time. At 12 h *p=0.031 G-15 [10 µM] vs. V, at 24 h ***p=0.000027 G-15 [1, 5 and 10 µM] vs V. At 48 h **p=0.0019 G-15 [1 µM] vs V and ***p=0.000021 G-15 [5 and 10 µM] vs V. The size effects for treatment η^2^=0.024, η^2^p=0.023. The results were analyzed by a two-way ANOVA test followed by the Bonferroni post hoc test; data are expressed as mean ± S.E.M (n=5).

**Supplementary Figure 5. G-15 effects on migration in the U87 cell line.** In a), representative images of wound closure at 0, 12, 24, and 48 h are shown for the treatments with G-15 at 1, 5, and 10 µM, and its V (DMSO 0.01%). b) Quantification of the wound area at the evaluated periods of time. At 12 hours **p=0.0025 G-15 [1 and 5 µM] vs. V. ***p=0.0000036 G-15 [10 µM] vs. V. At 24 hours the statistical differences were observed at 12 hours (**p=0.0012 and p=0.0003) were found, finally at 48 hours a statistical difference of *p=0.016 G-15 [10 µM] vs. V was found. The size effects for treatment were found η^2^=0.016, η^2^p=0.016. The results were analyzed by a two-way ANOVA test followed by a Bonferroni post hoc test; data are expressed as mean ± S.E.M (n=5).
